# A Lattice Distortion Theory for Promotor Containing Clathrate Hydrates

**DOI:** 10.1038/s41598-020-66776-2

**Published:** 2020-06-15

**Authors:** Niraj Thakre, Amiya K. Jana

**Affiliations:** Energy and Process Engineering Laboratory, Department of Chemical Engineering, Indian Institute of Technology, Kharagpur, 721 302 India

**Keywords:** Chemical engineering, Natural gas

## Abstract

A lattice distortion theory for promotor containing clathrate hydrates is formulated using the statistical thermodynamics based model of van der Waals and Platteeuw in association with the ab initio quantum mechanics to compute the cavity potentials. Despite of high degree of lattice distortion anticipated for large and polar molecules of liquid promotors, their variable lattice energy concept is unreported. With this intention, we estimate the lattice stabilization energy from spin-component scaled second order Møller-Plesset (SCS-MP2) perturbation theory applied with the augmented correlation-consistent polarized double zeta valence (aug-cc-pVDZ) basis set. Implementing this to compute cavity potential for different promotors, the reference properties of hydrates are harvested by regressing against the phase equilibrium conditions of their binary hydrates with methane. Our study confirms the exponential relation of reference chemical potential difference with van der Waals volume of the promotors. Moreover, using the excess Gibbs free energy theory, the higher order distortions for the multiple guests are captured. The proposed lattice distortion theory is attested with phase equilibrium conditions of eight promotors containing clathrate hydrate systems, namely propylene oxide, acetone, tetrahydrofuran, pyrrolidine, iso-butanaldehyde, cyclopentane, furan and thiophene, all having methane as a co-guest.

## Introduction

Natural gas hydrate (NGH) has attracted the interest for its occurrence as an energy resource which is believed to have up to 15,000 billion tons of carbon compared to 5,000 billion tons of all other sources of oil and gas around the globe^1^. Stability of the hydrates is favoured by the low temperature and high-pressure conditions, which is eventually accountable for its occurrence in permafrost and marine continental slope^2,3^. This typical crystalline compound is formed by hydrogen-bonded water molecules that entrap the small gas in a cage-like structure^4^. In addition to the gaseous guests, the light organic compounds, e.g. ethers, ketones, aldehydes, refrigerants and sulphur containing compounds, are capable to form more stable hydrates that are named as clathrate hydrates in general. Moreover, if mixed in a proper proportion with water, they can form the binary hydrates at mild conditions allowing the small gaseous components to co-guest them. This apart, some organics, namely methanol and ethylene glycol, inhibit the hydrate formation and therefore increase the formation pressure. With the ability to control the formation conditions, the promotors and inhibitors are potential candidates in flow assurance, NGH energy production and hydrate based applications of desalination and gas storage^5,6^. The phase behaviour of these hydrates is quite different from each other that needs the molecular level understanding to predict their bulk level properties. Although thermodynamics of the clathrate hydrate formation is well developed in the past four decades, the stability studies of the promotor containing hydrates and their phase behaviour are one of the current attentions^7^. The hydrate cages have pentagonal dodecahedra (5^12^) cavity as the basic building block which recombine in such a way to form different clathrate hydrate structures, amongst the sI, sII and sH are mostly found in nature^2^.

Investigating free energy for the noble gas hydrates of increasing molecular size of the guest demonstrates that their large size favours stable hydrate lattice by contributing more potential energy^8^. On the other hand, the large size of the guest molecule distorts the hydrate lattice that is evident from analysing the adsorption energies of ethane, propane and isobutane in different cages of the hydrate^9^. To model this phenomenon, the statistical thermodynamics approach of van der Waals and Platteeuw^10^ is available that is originally formulated on the basis of a constant crystal lattice assumption. This leads to a unique set of reference chemical properties in the calculation of the change in chemical potential of water in empty hydrate and bulk phase^11^. A modification for incorporating the lattice distortion is first proposed by computing the reference properties for individual guests having a diameter in the range of 4.10 Å for nitrogen to 6.50 Å for isobutane^12^. The change in host-host interaction energy in the perturbed and unperturbed lattice is added to the original chemical potential difference to update the new reference chemical potential difference. A constant pressure molecular dynamic simulation discerns the stretching of the lattice by the introduction of guests and their effect on macroscopic properties^13^. Lee and Holder^14^ documented the lattice distortion as a linear relation between the cavity radius and reference chemical potential difference (RCPD). The variable RCPD for gaseous guest is expressed as a two-parameter exponential function of Kihara hard core radius for both sI and sII type of clathrate hydrates^14^. The effect of secondary distortion for mixture hydrates is addressed by the probabilistic interaction among the neighbour guests^15^. An iterative procedure updates the RCPD with cavity radius using the relationship proposed by Zele *et al*.^13^ and reduces the errors in estimating the dissociation pressures for various sI and sII hydrates^16^. Meanwhile, Klauda and Sandler^17^ propose a fugacity-based model that claims that the variable lattice energy is incorporated in the empty hydrate phase fugacity term that is fitted to an empirical model. A lattice distortion model based on their approach by Martin and Peters^18^ expresses a linear relationship between distortion chemical potential and ratio of guest to cavity, however, no clear relationship is observed with the diameter of the guest.

Note that the cavity potentials used in calculating the RCPD in all abovementioned methods are either derived from molecular dynamics or second virial coefficient and viscosity data. A sensitivity analysis performed by Cao *et al*.^19^ on the uncertainties in estimating the reference chemical potential from the experimental data reports its substantial effect on the phase equilibrium predictions. Therein, as compared to the cavity potentials regressed to experimental hydrate data, the ab initio derived potentials produce relatively small statistical errors^19^. Nonetheless, the ab initio energy landscape for methane molecule rotating in the dodecahedra cages clearly captures the anisotropic potentials^20^. With this fact, most of the subsequent studies on the lattice distortion models adopt ab initio methods for estimating the cavity potentials. Lee *et al*.^15^ reported the RCPD for a couple of binary hydrates considering the excess Gibbs potential and an ab initio derived cavity potential. However, they do not claim any relation of the computed RCPD with the guest dimensions. In this light, Garapati and Anderson^21^ propose an entropy based lattice distortion model that differentiates the minimum lattice energy configurations for differently sized guests. With this, they obtain an exponential relationship of the reference properties with the diameter of the guest. The model predicts the phase equilibrium of single and binary hydrates without any adjustable parameters. As far in our knowledge, the lattice distortion is only documented for the small gaseous guest molecules having a diameter less than 6.5 Å (isobutane). However, the distortion is more dominant for the larger guests, i.e. liquid promotors and inhibitors, having a diameter up to 7.38 Å for cyclohexane. As the degree of lattice distortion is very high in case of liquid promotors, the reference properties are expected to vary in a significant amount and thus, the assumption of constant lattice energy upon encapsulation of the guests is highly violated. Consequently, despite of extensive experimental studies on the sII-type hydrate with liquid promotors available in literature, the phase equilibrium models for most of them are not reported. The possible reason is the absence of accurate cavity potential parameters for the promotor containing clathrate hydrates. The existing ab initio methodologies in calculating the cavity potential are designed and validated only for the small guest molecules and are not suitable for the large molecules like hydrate promotors. With this research gap, we propose a novel lattice distortion formulation for hydrate promotors.

Addressing this crucial issue, we introduce a computationally feasible ab initio technique for computing cavity potential with reasonable accuracy for the liquid promotors. The methodology is attested with the experimental Raman spectroscopic derived cage occupancies of promotor containing hydrates^22^. We aim to derive a lattice distortion model for these hydrates and consequently predict the phase equilibrium for the same. The cavity potentials for a series of binary hydrates of methane with sII type of promotors are estimated using the ab initio methodology. This leads to the direct estimation of the chemical potential difference between the empty and filled hydrate. For phase equilibrium calculations, this quantity is equated to the chemical potential difference between the empty hydrate and liquid phase. The nonideality in vapor and liquid phases are explained with the modified Patel-Teja equation of state^23^ (PT-EoS) and modified universal quasi-chemical functional-group activity coefficient^24^ (UNIFAC) models. This technique leads to the independent calculations for all properties and leaves behind the RCPD to be fitted to the hydrate data. This ensures that the RCPD calculated in this method represents the sole effect of lattice distortion caused by enclathration of the promotors in the clathrate hydrate cages.

In this contribution, a feasible quantum mechanical ab initio technique is designed to compute the cavity potential for liquid promotors with reduced computation time and without compromising in accuracy. The SCS-MP2 calculations are performed using the aug-cc-pVDZ basis set, while the accuracy of the complete basis limit is achieved by systematically analysing the basis set superposition (BSSE) and completeness (BSCE) errors. The cumulative effect of five-dimensional interactions of the guests with the surrounded water molecules is evaluated. This offers an accurate cavity potential that is used to estimate the reference chemical potential difference between hydrate and liquid water phase, i.e. extent of lattice distortion.

## Theory

Ab initio methodology for the cavity potential calculation of promotor containing clathrate hydrates is segmented into two sections: firstly, the generation of the potential energy surface (PES) grid; secondly, the selection of QM method and basis sets. We present a scheme to construct the gird points similar to Cao and coworkers^25^ with necessary modifications regarding the asymmetry of the guest molecules. Next, a new scheme for feasible QM method is described without compromising in the accuracy.

### Grid generation for potential energy surface

The cell potential is derived from the ab initio potential energy surface drawn for the interaction of water and promotor molecule. According to its spatial orientations in the hydrate cage, the position of the guest molecule can be defined by six degrees of freedom; that are the spherical coordinates of the centre of mass of guest molecule with respect to the oxygen molecule of water $(r,\theta ,\phi )$ and the coordinates of all constituting atoms of the guest with its centre of mass as origin $(r{\prime} ,\theta {\prime} ,\phi {\prime} )$.

**Figure 1:**
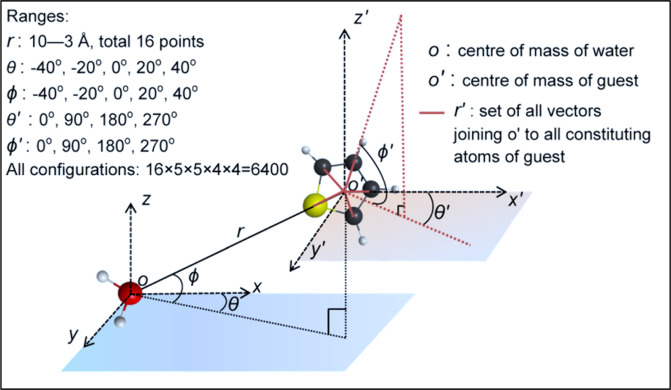
Guest-water dimer configuration is represented with five degrees of freedom. The ranges of the coordinates are determined by the geometrical constraints of the hydrate cavity.

The set of radial distances ($R{\prime}$) of all atoms of the guest molecule is fixed for constant geometry that results in five degrees of freedoms. The constraint imposed on the angles $\theta$ and $\phi$ is [–40, 40] that accords to its location in the cavity such that it is not much close to the cage wall. The spacing for the radial distance ($R$) of centre of mass of guest molecule with respect to the oxygen in water is set denser near the cage wall to arrest its stiff nature in repulsive region. On revolving the guest around the water molecule, this scheme generates 6400 nodes (Fig. 1) of PES grid. An arithmetical average of the energies computed at each radial distance ${E}_{avg}(r)$ results in the averaged cavity potential that can be fitted to suitable potential models.

1 $${E}_{avg}(r)=(1/400)\times \sum _{{\rm{all}}\,{\rm{angles}}}E(r,\theta ,\phi ,\theta {\prime} ,\phi {\prime} )$$

### QM methods and basis sets

Herein, we provide the QM method and basis set utilized in this work. The QM methods solves the electronic Schrödinger equation, for which the basic Hartree-Fock (HF) method is based on a mean field approximation that averages all the electronic interactions into a single effect. The Møller-Plesset perturbation theory (MPPT) incorporates electronic correlations to the Hartree-Fock, in which the ${n}^{{\rm{th}}}$ order of theory (${\rm{MP}}n$) corresponds to energy calculation up to ${(2n+1)}^{{\rm{th}}}$ order. While, the basis sets discretise the Schrödinger equation into the readily solvable algebraic equations. A comparison of the proposed and existing methodologies to compute the cavity potential is presented in Table 1. The first methodology is established by Cao *et al*.^25^, in which they used MP2/6–31 + +G(2d,2p) level of theory to compute potential energies for a total of 18000 orientations for a methane-water dimer. For improving the accuracy, they performed a high-level MP2/cc-pVQZ at 98 selected grids from modified Plackett–Burman design to capture the effect of high-level theory to each angular degree of freedom. The subsequent studies are reported on estimating cavity potential of the methane, argon and CO_2_ hydrates used MP2 level of theory with sufficiently large basis sets^26^. However, no estimations are reported for the hydrate promotors having large molecular size.

**Table 1:** Comparison of proposed and existing ab initio methodologies applied to estimate cavity potential.

Reference	QM method	Basis set	Guest molecule
Cao *et al*.^25^	MP2	6–31 + + G(2d,2p)	CH_4_
	MP2	cc-pVQZ	CH_4_
Klauda and Sandler^39^	MP2	6–31 + + G(3d,3p)	C1-C3, CO_2_
Anderson *et al*.^26^	MP2	aug-cc-pvQZ	CH_4_, Ar
Sun and Duan^40^	MP2	N.A.	CH_4_, CO_2_
Velaga *et al*.^41^	MP2	aug-cc-pvTZ	CO_2_
This work	SCS-MP2	aug-cc-pvDZ with complete basis set extrapolation	CH_4_ and promotors^#^

N.A. stands for Not Available, ^#^Promotor molecules include propylene oxide, acetone, tetrahydrofuran, pyrrolidine, iso-butanaldehyde, cyclopentane, furan and thiophene.

As the required computation time is considerably high for large molecular system of hydrate promotor used in the present study (say >15 atoms), this motivates us to design the ab initio methodology that is closely accurate to the coupled cluster (CC) and take computation time comparable to MPPT. In this light, we propose the spin-component scaled second order Møller-Plesset perturbation theory (SCS-MP2) by Antony and Grimme^27^. The spin-component scaled Møller-Plesset, SCS-MP2 is applied to estimate the non-covalent interaction energies of liquid organic promotors that has potential applications where the highly accurate CCSD(T) calculations cannot be performed. However, there are local correlation theories^28^ developed in recent years, e.g. domain-based local pair-natural orbital (DLPNO)-CCST(T)^29,30^ and local natural orbital (LNO)-CCSD(T)^31^, that can routinely compute the CCSD(T) level energies. The SCS-MP2 technique modifies the second order electronic correlation (${E}^{C}$) by scaling the double excitations of electron pair in parallel (${E}^{P}$) and antiparallel (${E}^{AP}$) spin as

2 $${E}^{C}={p}_{S}{E}^{P}+{p}_{T}{E}^{AP}$$

Here, the scaling parameters ${p}_{S}$ and ${p}_{T}$ have default values of 6/5 and 1/3, respectively. The basis sets discretise the Schrodinger equation into the readily solvable algebraic equations. The Dunning’s basis sets are designed such that the post-Hartree-Fock calculations converge systematically to the complete basis set (CBS) limit. This is utilized to correct the computed energies by analysing the basis set convergence (BSCE) and superposition (BSSE) errors. Let us take dimer of two molecules *A* and *B*. Using the counterpoise theory, the uncorrected ($\Delta {E}_{AB}^{{\rm{raw}}}$) and corrected ($\Delta {E}_{AB}^{{\rm{CP}}}$) energies are calculated in the following way

3 $$\Delta {E}_{AB}^{{\rm{raw}}}={E}_{AB}^{AB}-{E}_{A}^{A}-{E}_{B}^{B}$$

4 $$\Delta {E}_{AB}^{{\rm{CP}}}={E}_{AB}^{AB}-{E}_{A}^{AB}-{E}_{B}^{AB}$$

where, the convention ${E}_{A}^{AB}$ represents the potential energy of molecule $A$ on the basis of $AB$ dimer. The difference of the counterpoise corrected and uncorrected energy gives the estimation of the BSSE as follows

5 $${\rm{BSSE}}=\Delta {E}_{AB}^{{\rm{CP}}}-\Delta {E}_{AB}^{{\rm{raw}}}$$

Another error, i.e. BSCE is estimated by extrapolating the energies recorded at the increasing basis sets. Observing the expected nature of the variation of the energy values, the following equation is expected to give best fit and estimation of energy at complete basis limit^27^.

6 $$\Delta {E}_{\mathrm{int}}^{{\rm{raw}}}=\Delta {E}_{\mathrm{int}}^{{\rm{CBS}}}+B{n}^{-3}$$

Here, $B$ is a constant and $\Delta {E}_{\mathrm{int}}^{{\rm{CBS}}}$ is the desired potential energy at CBS. The symbol $N$ represents the cardinal number that holds $n$=2, 3, 4, 5 and so on, values for aug-cc-pV*n*Z type basis sets. In this way, the BSCE is the difference of counterpoise corrected and CBS energy

7 $${\rm{BSCE}}=\Delta {E}_{AB}^{{\rm{CP}}}-\Delta {E}_{AB}^{{\rm{CBS}}}$$

The can be estimated by the collective effects of these two errors as depicted in the following equation

8 $$\Delta {E}_{AB}^{{\rm{CBS}}}=\Delta {E}_{AB}^{{\rm{raw}}}+w\times {\rm{BSSE}}$$

Here, $w=1-{\rm{BSCE}}/{\rm{BSSE}}$ is the Pauling point counterpoise weight that is multiplied to the BSSE and the resultant is to be added to the raw interaction energies to get $\Delta {E}_{AB}^{{\rm{CBS}}}$ value. This value is calculated for a total of 6400 orientations of the guest-water dimer for the cavity potential used to calculate the hydrate phase nonidealities.

### Hydrate phase

The hydrate phase equilibrium occurs when the change in the chemical potential difference of water between the filled and empty hydrate ($\Delta {\mu }_{w}^{\beta -H}$) equals to the difference between empty hydrate and liquid phase ($\Delta {\mu }_{w}^{\beta -L}$),

9 $$\Delta {\mu }_{w}^{\beta -H}=\Delta {\mu }_{w}^{\beta -L}$$

where, $\beta$, $H$ and $L$ represent the empty hydrate, filled hydrate and liquid phases. The hydrate phase is a thermodynamically constrained solid-like state, in which a non-stoichiometric amount of the guest can hold the water molecules in a form of the crystalline lattice structure. The question of how much guest is needed to be fractionally occupied in water cavities can be answered by statistical thermodynamics. In this light, van der Waals and Platteeuw^10^ developed a model for estimating the change in chemical potential in empty and filled hydrate ($\Delta {\mu }_{w}^{\beta -H}$) using fractional occupancy (${\theta }_{ij}$) as

10 $$\Delta {\mu }_{w}^{\beta -H}=-\,RT\sum _{i}{\upsilon }_{i}\,\mathrm{ln}(1-\sum _{j}{\theta }_{ij})$$

Here, $i$ and $j$ are indices for the cavity and guest. The number of cavities per water molecule (${\upsilon }_{i}$) for sII hydrate are 1/23 and 3/23 small (5^12^) and large (5^12^6^4^) cages, respectively. The reaction of converting empty cages into filled cages is governed by Langmuir constant (${C}_{ij}$) and the fugacity (${f}_{j}$)

11 $${\theta }_{ij}=\frac{{C}_{ij}{f}_{j}}{1+\sum _{j}{C}_{ij}{f}_{j}}$$

The fugacities of the component in vapor and liquid phases are calculated using the modified PT equation of state^23^. The Langmuir constant is a measure of cavity stabilization by the effective guest-water interaction. This is estimated by volume integration of the Boltzmann weighted averaged cavity potential as

12 $$C=\frac{4\pi }{kT}{\int }_{0}^{\infty }\exp (-\omega (r)/kT){r}^{2}dr$$

Here, $\omega$ is the averaged cavity potential for which we have developed the ab initio methodology. This can be represented by a three-parameter Kihara potential model^3^.

13 $$\omega (r)=4\varepsilon \left[{\left(\frac{\sigma -a}{r-a}\right)}^{12}-{\left(\frac{\sigma -a}{r-a}\right)}^{6}\right]$$

This generalized formulation is modified for the specific hydrate cavities as follows

14 $$\omega (r)=2z{\prime} \varepsilon \left[\frac{{\sigma }^{12}}{{R}^{11}r}\left({\delta }^{10}+\frac{a}{R}{\delta }^{11}\right)-\frac{{\sigma }^{6}}{{R}^{5}r}\left({\delta }^{4}+\frac{a}{R}{\delta }^{5}\right)\right]$$

15 $${\delta }^{N}=\frac{1}{N}\left[{\left(1-\frac{r}{R{\prime} }-\frac{a}{R{\prime} }\right)}^{-N}-{\left(1+\frac{r}{R{\prime} }-\frac{a}{R{\prime} }\right)}^{-N}\right]$$

where, $R{\prime}$ and $z{\prime}$ represent the radius and coordination number of the cavity, respectively. The coordination number is the count of water molecules per hydrate cavity. The Kihara potential parameters $\sigma$, $\varepsilon$ and $a$ are obtained by fitting Eq. (13) to the angle averaged ab initio energies ${E}_{avg}(r)$ estimated in Eq. (1).

For hydrate equilibrium calculation, the change in chemical potential in empty and filled hydrate is equated to the change in the empty hydrate and liquid water ($\Delta {\mu }_{w}^{\beta -L}$). For estimation of the latter quantity, the Holder’s equation^32^ is applied as

16 $$\frac{\Delta {\mu }_{w}^{\beta -L}}{RT}=\frac{\Delta {\mu }_{w}^{\beta -L,o}}{R{T}_{o}}-{\int }_{{T}_{o}}^{T}\frac{\Delta {h}_{w}}{R{T}^{2}}dT+{\int }_{0}^{P}\frac{\Delta {V}_{w}}{RT}dP-\,\mathrm{ln}({\gamma }_{w}{x}_{w})$$

Here, the term $\Delta {\mu }_{w}^{\beta -L,o}$ indicates the reference chemical potential difference, while the other three terms correct the chemical potential for operating temperature, pressure and activity, respectively. The change in specific heat ($\Delta {h}_{w}$), molar volume difference of the water in the hydrate and liquid phase ($\Delta {V}_{w}$), and activity of water (${\gamma }_{w}$) are used for these corrections. The heat and volume corrections for the clathrate hydrates yield following expressions:^33^

17 $$\Delta {h}_{w}=\Delta {h}_{w}^{o}+{\int }_{{T}_{o}}^{T}[-38.12+0.141(T-{T}_{o})]dT$$

18 $$\Delta {V}_{w}=\Delta {V}_{w}^{o}+6.695\times {10}^{-15}({{\rm{m}}}^{3}{{\rm{mol}}}^{-1}{{\rm{Pa}}}^{-1})P$$

The values for $\Delta {h}_{w}^{o}$ and $\Delta {V}_{w}^{o}$ at standard point are −5202.2 J·mol^−1^ and 5.0 cm^3^·mol^−1^ for sII type hydrates, respectively^33^. For the estimation of activity of water altered by presence of promotors, the modified UNIFAC^24^ model is used.

### Lattice distortion model formulation

Holder *et al*.^32^ developed a method for estimating a single set of reference properties for sI hydrates irrespective of the nature of guests. The method is updated by Lee and Holder^14^ for calculation of variable reference properties for both the pure and mixture hydrates^34^. Combining Eqs. (9) and (16),

19 $$\frac{\Delta {\mu }_{w}^{\beta -H}}{RT}=\frac{\Delta {\mu }_{w}^{\beta -L,o}}{RT}-{\int }_{{T}_{o}}^{T}\frac{\Delta {h}_{w}}{R{T}^{2}}dT+{\int }_{0}^{P}\frac{\Delta {V}_{w}}{RT}dP-\,\mathrm{ln}({\gamma }_{w}{x}_{w})$$

Rewriting Eq. (17) in general form, we have

20 $$\Delta {h}_{w}=\Delta {h}_{w}^{o}+{\int }_{{T}_{o}}^{T}\Delta {C}_{{p}_{w}}dT$$

Rearranging Eq. (19) and adopting the $\Delta {h}_{w}$ from Eq. (20), the following equation is obtained for reference properties calculation.

21 $$\frac{\Delta {\mu }_{w}^{\beta -H}}{T}+{\int }_{{T}_{o}}^{T}\left\{\frac{1}{{T}^{2}}{\int }_{{T}_{o}}^{T}\Delta {C}_{{p}_{w}}dT\right\}dT-{\int }_{0}^{P}\frac{\Delta {V}_{w}}{T}dP+R\,\mathrm{ln}({\gamma }_{w}{x}_{w})=\frac{\Delta {\mu }_{w}^{\beta -L,o}}{{T}_{o}}+\Delta {h}_{w}^{o}\left[\frac{1}{T}-\frac{1}{{T}_{o}}\right]$$

Let us consider the left-hand side as $Y$ and $[1/T-1/{T}_{o}]$ as $X$. Eventually, Eq. (21) can be written as

22 $$Y=SX+I$$

When $Y$ is plotted against $X$, one can obtain the slope ($S$) and intercept ($I$) that lead to the values of reference properties.

23 $$\Delta {\mu }_{w}^{\beta -L,o}=I\times {T}_{o}$$

24 $$\Delta {h}_{w}^{o}=S$$

Equations (23) and (24) produce the reference properties for the specific guests in pure and mixture hydrates. The combined effect of dissimilar guests on reference properties can be explained with the excess Gibbs free energy-based approach^15^. For the binary mixture, if the value is known for one component, the same for another can be computed using the hydrate molar concentration weighted correlation as follows,

25 $$\Delta {\mu }_{w}^{mix,o}=\Delta {\mu }_{w}^{m,o}{Z}_{1}+\Delta {\mu }_{w}^{p,o}{Z}_{2}+{Z}_{1}{Z}_{2}+[{A}_{12}+{B}_{12}({Z}_{1}-{Z}_{2})]$$

26 $$\Delta {h}_{w}^{mix,o}=\Delta {h}_{w}^{m,o}{Z}_{1}+\Delta {h}_{w}^{p,o}{Z}_{2}+{Z}_{1}{Z}_{2}+[{A{\prime} }_{12}+{B{\prime} }_{12}({Z}_{1}-{Z}_{2})]$$

Here, o in the superscript of $\Delta {\mu }_{w}^{mix,o}$ stands for its value at reference point (273.15 K and 0 MPa). The symbols ${Z}_{1}$ and ${Z}_{2}$ represent the hydrate phase compositions of component 1 and 2, respectively. The parameters ${A}_{12}$ and ${B}_{12}$ stand for the intreaction between component 1 and 2, respectively. The superscripts *m* and *p* denote the methane and promotor molecules, respectively. The first two terms in Eqs. (25) and (26) account for the primary lattice distortion, whereas the third and fourth terms account for the higher order distortions.

### Simulation algorithm and model identification

The quantum mechanical simulations are performed in GAMESS-US^35^ (version: 2018-R1-pgi-mkl) for evaluating the guest-water interaction energies. The individual molecules are optimized using SCS-MP2 theory and aug-cc-pVDZ basis set. The initial configuration of the guest-water dimer is chosen in such a way that their dipole moments coincide with each other. A total of 6400 input files are generated using a MATLAB^®^ code for the different orientations of the guest with respect to the water molecule, as described in Fig. 1. For counterpoise energy calculation, we set zero point charge for each molecule separately to generate other two sets of input files. Single point energies are computed for all three sets of input files with the same level of theory with which the individual molecules are optimized. The counterpoise corrected ($\Delta {E}_{AB}^{{\rm{CP}}}$) and uncorrected ($\Delta {E}_{AB}^{{\rm{raw}}}$) energies are calculated using Eqs. (3) and (4). The energies are arithmetically averaged at each radial position and fitted to the Kihara potential function to obtain the collision diameter ($\sigma$) and energy well depth ($\varepsilon$).

Subsequently, the reference properties are estimated in Holder’s equation for the change in chemical potential and enthalpy during phase change from water to empty hydrate at the reference point. The cavity potential parameters are employed to estimate the Langmuir constant (${C}_{ij}$) using Eq. (12). The fugacity of guest in the equilibrium phases estimated using the modified equation of state model presented in Methods section. The Langmuir constant and fugacity of guest quantify the cage occupancy, ${\theta }_{ij}$ using Eq. (11) and subsequently, the filled-empty hydrate chemical potential difference is estimated using Eq. (10). This is equated to the empty hydrate-water chemical potential difference in Holder’s equation presented in Eq. (16). The change in the activity of water in the liquid phase due to presence of promotor is estimated using the modified UNIFAC model featuring in Methods section. In this way, the experimental hydrate formation conditions are applied to Eq. (21) and linearly fitted the cumulative effect of hydrate stabilization and destabilization factors to the operating temperature. The intercept and slope of this line are saved as reference chemical potential and enthalpy differences, respectively.

## Results

### Cavity potential and hydrate cage occupancy

**Figure 2:**
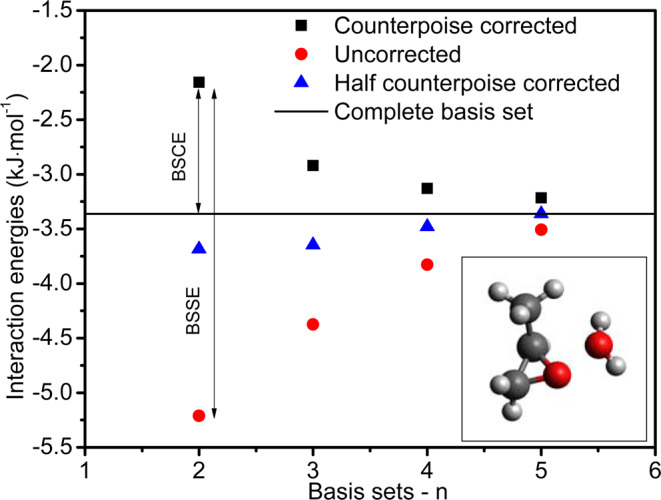
Estimation of Pauling-point correction factor for propylene oxide-water pair. The configuration of the pair is given in inset.

The estimation of BSCE and BSSE is presented for propylene oxide in Fig. 2. Four different Dunning’s basis sets, namely aug-cc-pVDZ, aug-cc-pVTZ, aug-cc-pVQZ and aug-cc-pV5Z having cardinal numbers *n* = 2, 3, 4 and 5, respectively, are utilized to compute CBS energy. By extrapolating the energies to complete basis set using Eq. 6, the value of Pauling-point correction factor is estimated as *w* = 0.60 for the configuration shown in inset of Fig. 2. This quantity holds value around 0.5 for different configurations and in this way, the overall effect validates the half-counterpoise method. Consequently, we choose this method for rest of the calculations.

**Figure 3:**
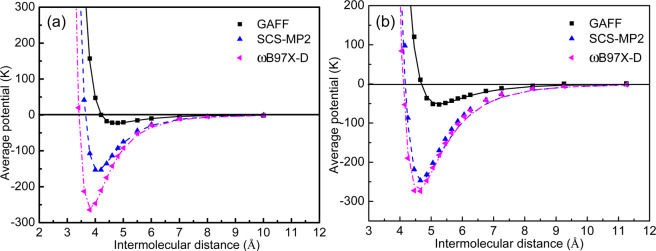
Comparison of different QM methods for (**a**) methane-water and (**b**) tetrahydrofuran-water systems. Scatter and line represent the computed energies and Kihara potential model fit, respectively.

**Figure 4:**
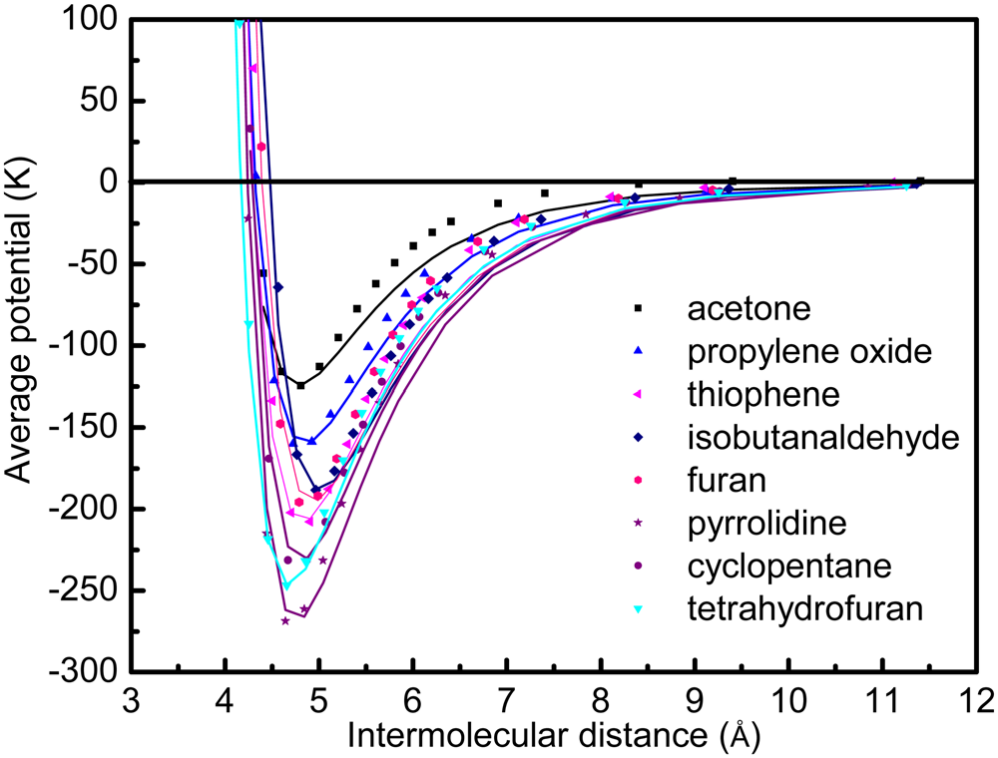
Cavity potential calculations for the clathrate hydrate promotors.

To examine the accuracy of SCS-MP2 method over other schemes, we compute the energies using SCS-MP2, general AMBER force field (GAFF) and density functional theory (ωB97X-D) for methane and tetrahydrofuran system at aug-cc-pvDZ basis set (Fig. 3). The potential energies obtained from these simulations are arithmetically averaged for the angular degrees of freedom and plotted against the intermolecular distances. The nature of the energy curves suggests that the cavity potential follows the typical Lennard-Jones 12-6 theory. Fitting the ab initio energies to Eq. (13), the estimated cavity potential parameter ($\varepsilon /k$, $\sigma$) for CH_4_ and THF using SCS-MP2, GAFF and ωB97X-D functional and resulting cage occupancies are presented in Table 2. With respect to the experimental values of cage occupancies, the percentage absolute error using ab initio (SCS-MP2) method is observed to be insignificant as compared to GAFF and ωB97X-D. Consequently, we recommend the SCS-MP2 theory for computing cavity potential of the promotor containing clathrate hydrates (Fig. 4).

**Table 2:** Comparison of potential parameters and their effect on cage occupancies for different QM methods.

Guest	Method	*ε*/*k* (K)	*σ*(Å)	${\theta }_{S}^{\mathrm{mod}}$^#^	${\theta }_{L}^{\mathrm{mod}}$^#^	$|{\theta }_{S}^{\mathrm{mod}}-{\theta }_{S}^{\exp }|$ × 100(%)^#^	$|{\theta }_{L}^{\mathrm{mod}}-{\theta }_{L}^{\exp }|$ × 100(%)^#^
CH_4_	GAFF	23.35	4.217	0.0007	0.0000	89.82	97.20
	SCS-MP2	153.75	3.641	0.9183	0.9718	1.93	0.02
	ωB97X-D	260.16	3.413	0.9999	0.9999	10.09	2.79
THF	GAFF	51.29	4.680	0.0000	0.0000	0.00	100.00
	SCS-MP2	246.75	4.166	0.0000	1.0000	0.00	0.00
	ωB97X-D	269.98	4.097	0.0000	1.0000	0.00	0.00

^#^Cage occupancies are estimated at 274.6 K and 3.21 MPa and compared with their experimental values (${\theta }_{S}^{\exp }$, ${\theta }_{L}^{\exp }$), which are (0.899, 0.972) and (0, 1), for CH_4_ and THF, respectively. The subscripts *S* and *L* stand for the small and large cages of the clathrate hydrate, respectively.

The cavity potential parameters for different promotors estimated at SCS-MP2/aug-cc-pVDZ level are reported in Table 3. The Kihara hard core radius is set as zero because it owns a negative value while fitting ab initio energies^18^. Analysing the potential curves, an important observation is made that the acetone promotes the methane hydrate formation despite of having lower energy well depth ($\varepsilon /k$ = 124.59 K) as it is able to make more stable hydrate structure sII than the sI hydrate of pure methane. The cavity potentials are estimated with varying the distance of guest from the cage wall, thus these generalized parameters are applicable to any size of the cage and hence to every available hydrate structures.

**Table 3:** Lennard-Jones potential parameters for the promotors estimated using ab initio method.

Promotors	*ε*/*k* (K)	*σ*(Å)
Acetone	124.59	4.255
Propylene oxide	159.83	4.319
Thiophene	207.75	4.295
Isobutanaldehyde	188.28	4.456
Furan	195.75	4.391
Pyrrolidine	268.63	4.239
Cyclopentane	231.61	4.283
Tetrahydrofuran	246.75	4.166

### Quantifying the extent of lattice distortion

**Figure 5:**
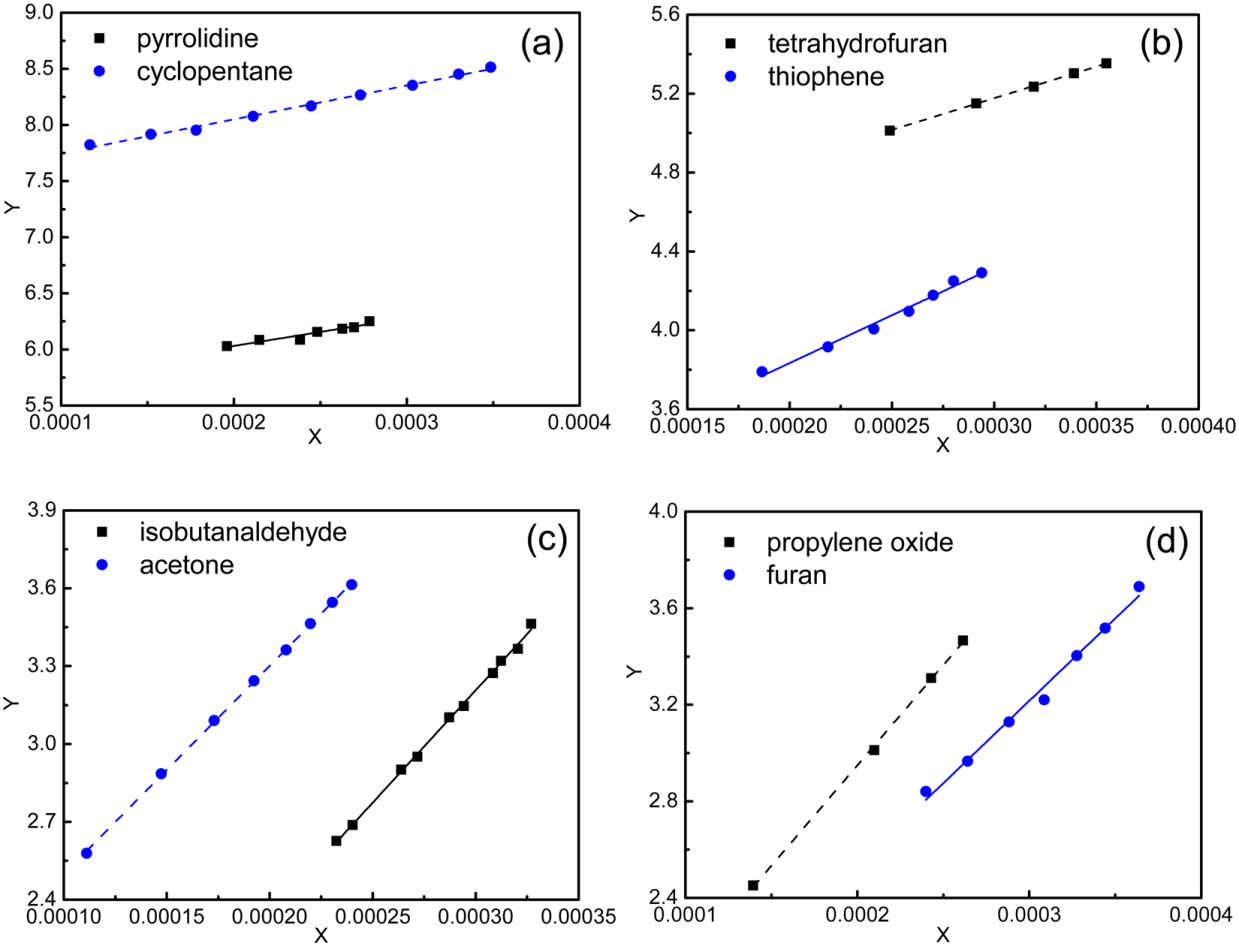
Reference properties calculation using Holder’s equation for the binary hydrates of methane containing promotors: (**a**) pyrrolidine and cyclopentane, (**b**) tetrahydrofuran and thiophene, (**c**) isobutanaldehyde and acetone, and (**d**) propylene oxide and furan.

Most of the studied sII hydrate formers are self-forming and they do not need the guest gas to form the hydrates. However, the phase equilibrium data for these promotors are mostly available as binary hydrates with methane as a co-guest. Consequently, the Holder’s equation produces directly the reference properties for binary hydrate ($\Delta {\mu }_{w}^{mix,o}$). The phase equilibrium data of these binary hydrates of promotors + methane are used to generate the X-Y plots (Eq. (22)), whose slope and intercept refer to the enthalpy and chemical potential difference at reference condition, respectively (Fig. 5). These values are reported in Table 4 for binary hydrates of all the eight promotors.

**Table 4:** Estimated mixed reference properties for promotor containing clathrate hydrates.

Guest species	$\Delta {\mu }_{w}^{\beta -L,o,mix}$ (J·mol^−1^)	$\Delta {h}_{w}^{o,mix}$ (J·mol^−1^)
Propylene oxide	351.30	−8313.6
Acetone	463.20	−8026.7
Tetrahydrofuran	1151.0	−3208.6
Pyrrolidine	1511.7	−2484.3
Isobutanaldehyde	166.00	−8663.5
Cyclopentane	2034.1	−3014.7
Furan	319.30	−6824.0
Thiophene	782.20	−4849.0

In case of mixture hydrates, existing lattice distortion models are formulated for the similar size of gaseous guests. They assume that the effect of distortion would be the same for all type of guests^14^. In the present case of promotor containing hydrates, the co-guest is methane which is smaller enough and in consequence, the lattice distortion effect is negligible as compared to the large molecule of promotor. In this light, we modified the theory taking into account the sole effect of lattice distortion by promotors. However, the methane-promotor interactions are considered to capture the higher order distortions. In this light, we adopted the Gibbs free energy based lattice distortion model^15,16^ that leads to the estimation of reference properties for the pure promotors as well as the interaction terms listed in Table 5.

**Table 5:** Estimated excess Gibbs free energy model parameters for hydrate promotors.

Guest species	*V*_vdW_ (Å^3^)	$\Delta {\mu }_{w}^{p,o}$	*A*_12_	*B*_12_	$\Delta {h}_{w}^{p,o}$	*A*_12_	*B*_12_
Propylene oxide	56.89	351.320	0.00700	−0.0090	−8320.73	−63.246	218.72
Acetone	66.61	473.719	−1529.9	−994.73	−8026.69	−0.012	1.1730
Tetrahydrofuran	74.19	1184.22	−154.76	98.5790	−3215.55	26.219	13.341
Pyrrolidine	76.40	1101.81	1493.89	1018.62	−2499.63	56.412	36.507
Isobutanaldehyde	77.99	166.046	4.63200	−6.0170	−8665.44	−190.81	258.426
Cyclopentane	82.70	2033.55	2.12300	0.42000	−3016.31	6.9760	−0.3780
Furan	58.01	319.342	0.01700	0.03300	−6824.22	0.5900	1.5980
Thiophene	67.73	774.605	27.31700	20.9520	−4852.73	13.504	10.039

**Figure 6:**
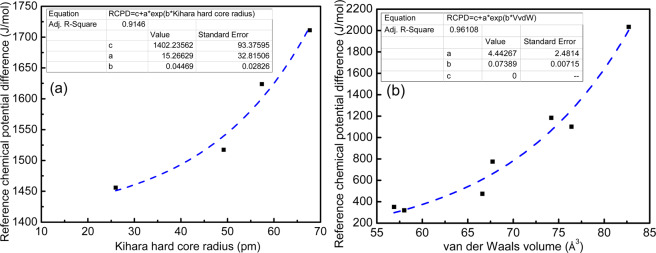
Lattice distortion model identification for (**a**) sI and (**b**) sII hydrates.

All the promotors are expected to follow the trend of decrease in hydrate formation pressures with increasing in their molecular sizes. In order to quantify the extent of lattice distortion for the promotor containing clathrate hydrates, we examined the variation of the calculated reference properties with respect to the size of the guests. For this purpose, we choose the van der Waals (vdW) volume of the promotor molecules calculated using the Bondi’s group contribution method^36^,

27 $${V}_{{\rm{vdW}}}=\sum {\rm{all}}\,{\rm{atoms}}\,{\rm{contributions}}-5.92{N}_{{\rm{B}}}-14.7{R}_{{\rm{A}}}-3.8{R}_{{\rm{NR}}}$$

where, the volume contributions of individual atoms considered in the present study are taken from literature^36^. The parameters ${N}_{{\rm{B}}}$, ${R}_{{\rm{A}}}$ and ${R}_{{\rm{NR}}}$ are the number of bonds present, and the number of aromatic and nonaromatic rings, respectively. The estimated vdW volumes for the promotors are shown in Table 5. The RCPD estimated using the ab initio potentials shows an exponential increment with the vdW volume of the promotor molecules (Fig. 6). This shows that the larger guest molecules destabilize the hydrate lattice more than the small guests despite of its high contribution to lattice distortion. A good exponential fit of the RCPD obtained with Kihara hard core radius for sI type of hydrate is expressed in Eq. (28). However, for the liquid phase promotors making sII hydrate, the better correlation of the computed RCPD is found with vdW volume of the promotor guest molecules as depicted in Eq. (29).

28 $${\rm{RCPD}}=1402.24+15.266\exp (0.04469a),\,{\rm{for}}\,{\rm{sI}}\,{\rm{hydrate}},$$

29 $${\rm{RCPD}}=5.45674\exp (0.07142\,{V}_{{\rm{vdW}}}),\,{\rm{for}}\,{\rm{sII}}\,{\rm{hydrate}},$$

where, $a$ is the Kihara hard core diameter in pm and ${V}_{{\rm{vdW}}}$ is in Å^3^. Equations (28) and (29) are the proposed lattice distortion models for the pure (sI) and promotor containing (sII) clathrate hydrates, respectively. This proposed lattice model for promotor containing hydrate uses vdW volume of the guest that applies to all categories of liquid sII hydrate formers.

Comparing to previous lattice distortion theories in Table 6, the proposed model shows an excellent correlation between the size of the guest and RCPD as compared to Lee and Holder^16^. For sI hydrate formers, correlating the Kihara hard core radius with the estimated RCPD is observed to improve slightly from 0.90 to 0.91. The two existing models of lattice distortion consider the guest size as Kihara hard core radius^14,16^ and guest diameter^21^ to correlate with the estimated RCPD. The smaller gaseous guests exhibit almost spherical shape, for which the molecular diameter is reasonable to quantify their sizes^21^. However, for the highly non-spherical shape of the large molecules of promotors studied in this work, the vdW volume represents realistic property. For example, the molecular diameter of THF is 5.90 Å^37^, which gives equivalent spherical volume of 107.5 Å^3^ ^37^, while the vdW volume is 74.19 Å^3^. This difference is attributed to the fact that the vdW volume is computed using the Bondi’s group contribution method^36^ that sums the volumes of individual atoms, whereas the equivalent spherical volume considers the whole molecule as a sphere. This makes the vdW volume as an effective property of non-spherical guest molecules. The coefficient of correlation *R*^2^ is improved for sII from 0.87 to 0.96. It should be noted that the previous studies only consider the nonpolar hydrate formers, while the present study investigates the polar liquid hydrocarbons. The maximum extent of lattice distortion reported in nonpolar category is 1887 J·mol^−1^ for isobutene, while it equals to 2034 J·mol^−1^ for cyclopentane in the liquid promotor category.

**Table 6:** Comparison with previous studies on lattice distortion models.

$${\rm{RCPD}}=\,c+a\exp (b\,\times {\rm{Guest}}\,{\rm{Size}})$$							
Guest size parameter	Type	*a*	*b*	*c*	*R*^2^	Guest diameter range (Å)	Reference
Kihara hard core radius	sI	133.3900	0.0213000	0	0.9058	4.1–6.1	Lee and Holder^14,16^
	sII	171.9100	0.0101000	0	0.8810	3.8–6.5	
Diameter	sI	1197.279	0.0010933	0	N.A.	N.A.	Garapati and Anderson^21^
	sII	974.0330	0.0264400	0	N.A.	N.A.	
Kihara hard core radius	sI	15.26629	0.044690	1402.24	0.9146	4.1–6.1	Proposed model
vdW volume	sII^†^	4.442670	0.073890	0	0.9610	4.3–7.4^†^	

^†^Liquid promotor containing hydrates for lattice distortion, N.A. Not available.

The hydrate formation and promoting mechanism can be analysed with the RCPDs shown in Fig. 6. The values of RCPD for sI hydrate are in the range of 1450–1700 J·mol^−1^, whereas the same for sII hydrates range from 200–2000 J·mol^−1^. This apparently shows that the extent of lattice distortion is more in case of sI hydrates than sII hydrates. This can be explained with the energy well depth that is observed to be greater for sII hydrate formers than the sI type. Thus, the energy contribution to stabilize the lattice is more in case of sII hydrates, which leads to lower down RCPD to 350 J·mol^−1^ in case of propylene oxide.

### Phase diagrams for promotor containing clathrate hydrates

**Figure 7:**
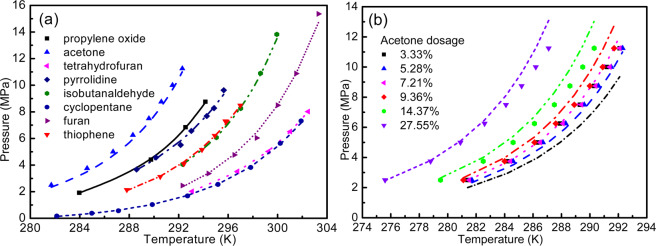
Phase equilibrium prediction of binary hydrates of methane with (**a**) 3% promotor dosasge for propylene oxide and 5.56% for rest of the promotors and (**b**) different acetone dosasge.

The proposed lattice distortion model is applied to predict the phase equilibrium for the binary hydrates of methane and promotors. The experimental phase equilibrium data is available for the stoichiometric proportions of the promotors to water ratio, i.e. 5.56%. Figure 7a depicts a close prediction of the hydrate formation pressures for a series of different promotor containing clathrate hydrate systems. The model performance is quantified in terms of percent absolute relative deviations (%AARD) and listed in Table 7. The AARD values hold a minimum value of 0.85% for four data points of methane + propylene oxide hydrate, while the value has a maximum value of 6.52% for 48 data points of methane + acetone hydrate. In the case of acetone, the phase predictions are shown for varying promotor dosage in Fig. 7b. The reasonable values of %AARD for the promotor containing clathrate hydrates attest the validity of the proposed lattice distortion model.

**Table 7:** AARD analysis for the phase equilibrium predictions using lattice distortion model.

Promotor	Number of data points	%AARD^†^
Propylene oxide	4	0.8506
Acetone	48	6.522
Tetrahydrofuran	32	3.541
Pyrrolidine	7	2.088
Isobutanaldehyde	15	3.318
Cyclopentane	9	3.553
Furan	7	2.181
Thiophene	7	2.077

^†^$$ \% {\rm{AARD}}=\frac{1}{n}\left(\sum \frac{|{P}^{\exp }-{P}^{\mathrm{mod}}|}{{P}^{\exp }}\right)\times 100$$.

Important findings of this study can be summarized as (i) formulating the ab initio methodology to compute cavity potential for promotor containing hydrates, (ii) implementing these potentials to estimate hydrate stabilization energies, (iii) regressing the reference properties against the stabilization energies and phase equilibrium conditions, (iv) obtaining a relation between reference properties with size of the promotor guests and finally, (v) using this lattice distortion model to predict the phase equilibrium conditions of promotor containing clathrate hydrates. Based on this proposed theory, it is clear that the vdW volume of the promotor molecules defines the extent of lattice distortion for hydrate promotors. As a future perspective, the study on vibrational frequencies for these promotors entrapped in the cavities can be a potential tool to envisage the relative stability of these hydrates using the loose-cage-tight-cage (LCTC) model^38^. Furthermore, this study can be extended to structure H type hydrates including the shape effects of hydrate cavities. The generalized approach reported in this work can provide a basic understanding for using the promotors in hydrate based applications.

## Discussion

The guest does distort the hydrate lattice and this phenomenon is more dominant in case of large molecule of hydrate promotors. In this view, we estimated the cavity potential for these hydrates using a novel ab initio technique featuring spin component scaled Møller-Plesset perturbation theory. This feasible and accurate methodology for computing intermolecular interaction energy is used for estimating lattice stabilization energy by encapsulation of the guest molecules. The reference chemical potential difference estimates a mixed effect of lattice distortion by differently sized molecules of methane and promotors. A negligible contribution of methane in distorting the lattice as compared to the large molecules of promotors is proposed as a modification in the existing Gibbs free energy model that is previously designed for mixtures of comparable molecular sizes. Moreover, for the liquid promotors, the van der Waals volume of the guest shows an excellent correlation coefficient of 0.96 while relating to the estimated reference chemical potential difference. This lattice distortion theory grounds the formulation of a generalized model for phase equilibrium predictions for the promotor containing clathrate hydrates.

## Methods

### Fugacity of guest

For the empty to filled hydrate phase reaction, the effective concentration of the guest is needed. We address this quantity in terms of fugacity of the guest compound in vapor and liquid phases calculated by modified Patel-Teja equation of state having the following form,

30 $$p=\frac{RT}{v-b}-\frac{a}{v(v+b)+c(v-b)}$$

This is a three parameter equation in pressure ($p$) and temperature ($T$). Here, *R* is a universal gas constant, and $a$, $b$ and $C$ have the following expressions,

31 $$a=\frac{{\varOmega }_{a}\alpha ({T}_{R}){R}^{2}{T}_{c}^{2}}{{P}_{c}}$$

32 $$b=\frac{{\varOmega }_{b}R{T}_{c}}{{P}_{c}}$$

33 $$c=\frac{{\varOmega }_{c}R{T}_{c}}{{P}_{c}}$$

where, ${T}_{c}$, ${P}_{c}$ and *T*_*R*_(=*T*/*T*_*c*_) are the critical temperature, critical pressure and the reduced temperature, respectively. In modified Patel-Teja equation of state, the term $\alpha ({T}_{R})$ is formulated as

34 $$\alpha ({T}_{R})=\exp \,\left[{H}_{1}{\left(1-\left\{\frac{T}{{T}_{C}}\right\}\right)}^{{H}_{2}}\right]$$

Here, the parameters (${H}_{1}$ and ${H}_{2}$) are available for various gaseous and liquid compounds in literature. In addition, these values can be calculated directly from the acentric factor as

35 $${H}_{2}=\frac{-0.2981-1.9574\varpi +0.1789{\varpi }^{2}}{0.4563+1.26\varpi -0.3928{\varpi }^{2}}+1.4563+1.26\varpi -0.3928{\varpi }^{2}$$

36 $${H}_{1}=\frac{0.4563+1.26\varpi -0.3928{\varpi }^{2}}{{H}_{2}}$$

While, the parameters Ω_*a*_, Ω_*b*_ and Ω_*c*_ have similar formulations as the original PT equation of state as,

37 $${\Omega }_{c}=1-3{\zeta }_{c}$$

38 $${\Omega }_{a}=3{\zeta }_{c}^{2}+3(1-2{\zeta }_{c}){\Omega }_{b}+{\Omega }_{b}^{2}+1-3{\zeta }_{c}$$

39 $${\Omega }_{b}^{3}+(2-3{\zeta }_{c}){\Omega }_{b}^{2}+3{\zeta }_{c}^{2}{\Omega }_{b}-{\zeta }_{c}^{3}=0$$

40 $${\zeta }_{c}=0.3272-0.0537\varpi -0.0147{\varpi }^{2}$$

The parameter ${\zeta }_{c}$ is the critical compressibility factor that can be directly computed from the acentric factor. In Eq. (39), Ω_*b*_ is the least positive real root. For estimation of compressibility factor ($z$), the modified PT equation of state can be rewritten as cubic in $z$,

41 $${z}^{3}+(C-1){z}^{2}+(A-2BC-{B}^{2}-B-C)z+(BC+C-A)B=0$$

in which, forms of $A$, $B$ and $C$ are as follows

42 $$A=\frac{ap}{{R}^{2}{T}^{2}}$$

43 $$B=\frac{bp}{RT}$$

44 $$C=\frac{cp}{RT}$$

After estimation of compressibility factor and three parameters of the Patel-Teja equation of state, the fugacity coefficient of the pure component can be readily calculated using the following expression:

45 $$\mathrm{ln}(\phi )=z-1-\,\mathrm{ln}(z-B)+\frac{a}{2RTN}\,\mathrm{ln}\left(\frac{z+M}{z+Q}\right)$$

where, $M$, $N$ and $Q$ have the following formulations

46 $$M=\left(\frac{b+c}{2}-N\right)\frac{p}{RT}$$

47 $$N={\left[bc+\frac{{(b+c)}^{2}}{2}\right]}^{-1/2}$$

48 $$Q=\left(\frac{b+c}{2}+N\right)\frac{p}{RT}$$

The fugacity of the component $i$ in the vapor phase can be estimated as

49 $${f}_{i}^{v}={\phi }_{i}{x}_{i}p$$

### Activity of water

The activity of water is influenced by the promotor introduced for hydrate formation. For this purpose, the modified UNIFAC model is adopted that expresses the molar excess Gibbs free energy (*G*^*E*^) as a summation of combinatorial (*G*^*C*^) and residual (*G*^*R*^) parts. For multicomponent systems, the activity coefficient can be expressed as,

50 $$\mathrm{ln}({\gamma }_{i})=\,\mathrm{ln}({\gamma }_{i}^{C})+\,\mathrm{ln}({\gamma }_{i}^{R})$$

where, ${\gamma }_{i}$ denotes the activity coefficient of component $i$. The combinatorial (${\gamma }_{i}^{C}$) part of the ${\gamma }_{i}$ has the following formulation

51 $$\mathrm{ln}({\gamma }_{i}^{C})=\,\mathrm{ln}\left(\frac{{\Phi }_{i}}{{x}_{i}}\right)+\frac{Z}{2}{q}_{i}\,\mathrm{ln}\left(\frac{{\theta }_{i}}{{\Phi }_{i}}\right)+{l}_{i}-\frac{{\Phi }_{i}}{{x}_{i}}\mathop{\sum }\limits_{j=1}^{N}{x}_{j}{l}_{j}$$

where, $j$ is the false index for summing over all components present in the system. The term ${l}_{i}$ is expressed as a combination of the area (${q}_{i}$) and volume parameters (${r}_{i}$) for species $i$ as

52 $${l}_{i}=\frac{Z}{2}({r}_{i}-{q}_{i})-{r}_{i}+1$$

53 $${q}_{i}=\sum _{k}{v}_{k}^{i}{Q}_{k}$$

54 $${r}_{i}=\sum _{k}{v}_{k}^{i}{R}_{k}$$

Here, $R$ and *Q* are the area and volume parameters for the functional groups with index $k$. The notation ${v}_{k}^{i}$ is the number of functional groups present of type $k$ in component $i$. The parameter *Z* denotes the coordination number of the system having a reasonable value of 10 and it is observed to have no substantial effect on activity calculation. The parameters ${\theta }_{i}$ and Ω_*i*_ are the molar weighed area and volume fractions that can be calculated as,

55 $${\theta }_{i}=\frac{{x}_{i}{q}_{i}}{\mathop{\sum }\limits_{j=1}^{N}{x}_{j}{q}_{j}}$$

56 $${\Phi }_{i}=\frac{{x}_{i}{r}_{i}}{\mathop{\sum }\limits_{j=1}^{N}{x}_{j}{r}_{j}}$$

The residual part of the activity coefficient accounts for the interaction among the groups present in the system. This is represented as,

57 $$\mathrm{ln}({\gamma }_{i}^{R})=\sum _{k}{v}_{k}^{i}(\mathrm{ln}\,{\Gamma }_{k}-\,\mathrm{ln}\,{\Gamma }_{k}^{i})$$

where,

58 $$\mathrm{ln}\,{\Gamma }_{k}={Q}_{k}\left[1-\,\mathrm{ln}(\sum _{m}{\Theta }_{m}{\psi }_{mk})-\sum _{m}\frac{{\Theta }_{m}{\psi }_{km}}{\sum _{n}{\Theta }_{n}{\psi }_{nm}}\right]$$

59 $${\Theta }_{m}=\frac{{Q}_{m}{X}_{m}}{\sum _{n}{Q}_{n}{X}_{n}}$$

60 $${X}_{m}=\frac{\sum _{i}{v}_{m}^{i}{x}_{i}}{\sum _{i}\sum _{n}{v}_{n}^{i}{x}_{i}}$$

The term ${\psi }_{mk}$ accounts for the interaction between the unlike-groups present in the system. The modified UNIFAC model calculates the ${\psi }_{mk}$ as follows,

61 $${\psi }_{mk}=\exp \left[-\frac{{a}_{mk}^{(1)}+{a}_{mk}^{(2)}T+{a}_{mk}^{(3)}{T}^{2}}{T}\right]$$

The values of the parameters ${a}_{mk}^{(1)}$, ${a}_{mk}^{(2)}$ and ${a}_{mk}^{(3)}$ can be found in literature.

## Data Availability

The simulation datasets generated during and/or analyzed during the current study are available from the corresponding author on reasonable request.

## Code availability

The code which was used in the findings of this study is available from the corresponding author upon reasonable request.
